# Comparison of effects of OSA treatment by MAD and by CPAP on cardiac autonomic function during daytime

**DOI:** 10.1007/s11325-015-1265-0

**Published:** 2015-10-13

**Authors:** Martin Glos, Thomas Penzel, Christoph Schoebel, Georg-Reiner Nitzsche, Sandra Zimmermann, Christopher Rudolph, Alexander Blau, Gert Baumann, Paul-Georg Jost-Brinkmann, Stefanie Rautengarten, Jan Christian Meier, Ingrid Peroz, Ingo Fietze

**Affiliations:** Center for Sleep Medicine, Charité—Universitätsmedizin Berlin, CCM-CC11, Charitéplatz 1, 10117 Berlin, Germany; Department of Cardiology and Angiology, Charité—Universitätsmedizin Berlin, CCM-CC11, Berlin, Germany; Department of Dentofacial Orthopedics, Orthodontics, and Pedodontics, Charité—Universitätsmedizin Berlin, CBF-CC3, Berlin, Germany; Department of Prosthodontics, Geriatric Dentistry and Craniomandibular Disorders, Charité—Universitätsmedizin Berlin, CBF-CC3, Berlin, Germany

**Keywords:** Mandibular advancement device, Obstructive sleep apnea, Cardiac autonomic function, Blood pressure, Continuous positive airway pressure, Heart rate variability, Baroreceptor sensitivity

## Abstract

**Purpose:**

The present study compared the effects of mandibular advancement therapy (MAD) with continuous positive airway pressure therapy (CPAP) on daytime cardiac autonomic modulation in a wide range of obstructive sleep apnea (OSA) patients under controlled conditions in a randomized, two-period crossover trial.

**Methods:**

Forty OSA patients underwent treatment with MAD and with CPAP for 12 weeks each. At baseline and after each treatment period, patients were assessed by polysomnography as well as by a daytime cardiac autonomic function test that measured heart rate variability (HRV), continuous blood pressure (BP), and baroreceptor sensitivity (BRS) under conditions of spontaneous breathing, with breathing at 6, 12, and 15/min.

**Results:**

Both CPAP and MAD therapy substantially eliminated apneas and hypopneas. CPAP had a greater effect. During daytime with all four conditions of controlled breathing, three-minute mean values of continuous diastolic BP were significantly reduced for both MAD and CPAP therapy. At the same time, selective increases due to therapy with MAD were found for HRV high frequency (HF) values. No changes were observed for BRS in either therapy mode.

**Conclusions:**

These findings indicate that both MAD and CPAP result in similar beneficial changes in cardiac autonomic function during daytime, especially in blood pressure. CPAP is more effective than MAD in eliminating respiratory events.

## Introduction

Obstructive sleep apnea (OSA) is the most common form of sleep-disordered breathing and affects about 5–7 % of the adult population [1]. Repetitive collapses of the upper airways during sleep lead to hypoxic phases with associated sleep fragmentation and sympathetic activation, the latter of which also persists during daytime with the resultant consequences [2]. OSA has accordingly been confirmed as an independent cardiovascular risk factor that may increase mortality if left untreated [3]. Continuous positive airway pressure (CPAP) therapy is manifestly an effective therapeutic approach. Many studies have demonstrated that CPAP therapy reduces cardiovascular risk and overall mortality rate if regularly employed by OSA patients [2–4]. CPAP therapy is accordingly recommended as the standard therapy in moderate to severe OSA. The CPAP effect can partly be explained by its reduction of sympathetic tone not only during the night but also during daytime [5].

In addition to investigation of blood pressure [4] and heart rate variability (HRV) [6], measurements of baroreceptor sensitivity (BRS) and of systolic blood pressure variability (SBPV) represent non-invasive methods of assessing autonomic activity [7]. Regular CPAP therapy improves these parameters during the night and daytime as evidence of reduction of sympathetic hyperactivation in OSA patients [5, 8]. Many trials have dealt with the effect of CPAP on HRV [9], and others have likewise reported the positive effect of CPAP on BRS [9, 10].

Unfortunately, many patients demonstrate limited adherence to CPAP therapy, and some patients decline this form of treatment. There is accordingly a need for alternative therapeutic approaches that are as effective as CPAP therapy—not only with respect to symptoms but also to the cardiovascular consequences of OSA.

Intraoral mandibular advancement devices (MAD) protrude the mandible during sleep to maintain upper airway patency [11]. A number of trials have shown comparable effects of MAD and CPAP in OSA patients with regard to symptoms such as daytime sleepiness and driving performance—but have also covered side effects, withdrawal rates, and usage time [12, 13]. Although MAD evidently does not reduce apnea-hypopnea index (AHI) by the same amount as CPAP [12–14], more favorable side effects, withdrawal rates, and usage time may be explained by better compliance and quality of life in patients who employ MAD compared with those who use CPAP [13]. This was also confirmed by other randomized controlled trials that investigated the effects of both therapies [15–21]. MAD has thus been proposed as alternative therapeutic approach for mild to moderate OSA, in accordance with existing guidelines [2, 22]. Some investigators have demonstrated subjective benefits of MAD for patients classified with severe OSA [13, 18, 23]. Studies of MAD therapy, however, are often not comparable. Furthermore, the few studies that until now have examined the effects of MAD therapy on cardiovascular parameters have arrived at contradictory findings. Gotsopoulus et al. [24] found a small reduction in diastolic blood pressure during daytime, whereas Andren et al. [25] reported improvement in systolic blood pressure. Phillips et al. [13] showed that regular use of neither CPAP nor MAD for 1 month can improve 24-h ambulatory mean blood pressure, whereas they are comparably effective with respect to daytime sleepiness and quality of life.

Little is known about the effect of MAD therapy on autonomic activity parameters. One study investigating daytime HRV parameters [26] found that 3 months of MAD therapy improved cardiac autonomic modulation. Dal-Fabbro et al. [27] investigated effects of 1-month MAD and CPAP therapy on HRV during sleep and found a number of beneficial changes for both therapies.

To date, none of the previous studies has specifically explored in parallel the effect of MAD on daytime blood pressure, HRV, BRS, and SBPV. There are likewise no systematic comparisons between MAD and CPAP for these parameters in the same patients.

The present study therefore aimed to evaluate the effect of MAD and CPAP with regard to cardiovascular parameters and autonomic activity in a two-period crossover design in which the patients were either randomized to the sequence MAD–CPAP (12 weeks of MAD followed by 12 weeks of CPAP) or the sequence CPAP–MAD (3 months of CPAP followed by 3 months of MAD).

## Subjects and methods

### Participants

Eighty-four patients with suspicion of OSA syndrome were asked to participate in the study. The study was approved by the local ethics committee of the university hospital Charité—Universitätsmedizin Berlin, and participants gave their written consent for participation.

Inclusion criteria were an AHI of ≥5/h and an age of ≥18 years. Patients with severe OSA (AHI >30/h) requiring treatment were included only if they did not demonstrate clear indication for CPAP such as a severe cardiovascular risk, e.g., myocardial infarction, stroke, atrial fibrillation, resistant hypertension, or heart failure. An essential element for inclusion of any patient was a clinical symptom complex, as well as suffering owing to lack of refreshing sleep.

Exclusion criteria were drug abuses, any medication intake that could influence sleep, any presence of sleep disorders other than OSA, any kind of specific medication for OSA in the patient’s case history, prior use of any form of PAP therapy, any prior pharyngeal surgery (UPPP, LAUP, or RFT) for OSA therapy, any psychiatric or neurological diseases previously known or arising during the study that could impair compliance, atrial fibrillation, any medication that could affect heart rate, craniomandibular disorders with restricted mobility of the lower jaw (especially restrictions to protrusion), acute to subacute dental treatment requirements (e.g., caries treatment), >8 stable natural teeth per jaw (with maximum average Periotest value per tooth <20), acute periodontal disease, class III dental relationship with anterior crossbite, participants in orthodontic retention for less than 6 months, and discontinuation of therapy or interruption of therapy for more than 1 week.

Participants who had taken part in a clinical pharmacological trial up to 4 weeks before entering the study were also excluded.

### Study procedure

Patients with reported symptoms of OSA were asked to participate in the study. After receiving written consent for participation in the study, they received a 6-channel ambulatory sleep apnea monitoring device (Embletta pds, Embla Inc., Broomfield, CO, USA), which included recording airflow, snoring, thoracic and abdominal efforts, oxygen saturation, leg movements, and body position. In addition to a physical examination, a general medical case history, and a specific sleep disorder case history, patients were asked to complete the form on the Epworth Sleepiness Scale (ESS) as well as the Insomnia Severity Index (ISI). A dental examination and screening for craniomandibular disorders (CMD) were performed by a dentist.

**Fig. 1:**
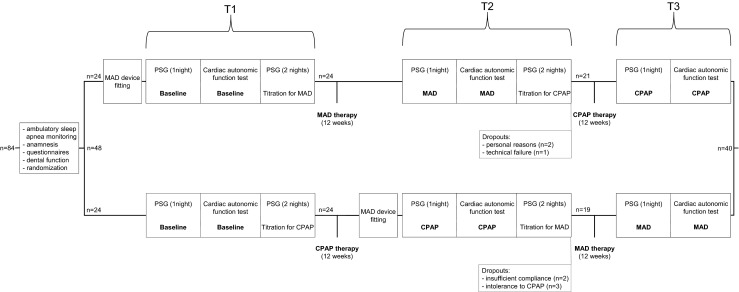
Flow diagram of the study: T1 at the beginning of therapy, T2 after 12 weeks of initial therapy, and T3 after another 12 weeks with the following therapy

If patients were eligible by the applied inclusion/exclusion criteria, they were randomized in a crossover fashion to either a) receiving MAD (*SomnoDent*^®^, Somnomed Europe AG, Zurich, Switzerland) as initial therapy for 12 weeks followed by CPAP (*REMstar Pro*, Philips Respironics, Murrysville, PA, USA) therapy for another 12 weeks (sequence group MAD–CPAP) or b) receiving CPAP as initial therapy for 12 weeks followed by a MAD therapy for another 12 weeks (sequence group CPAP–MAD). The entire study protocol is illustrated in Fig. 1.

If patients had been randomized to initially receive MAD therapy, the MAD was individually produced and fitted to the patient 1–2 weeks prior to the beginning of the therapy (T1) by the manufacturer (Somnomed Europe AG, Zurich, Switzerland) and by a dentist. At T1 in both treatment arms, patients were investigated by polysomnography (PSG) for three consecutive nights without gap. The first night served as baseline PSG, and the subsequent two nights were titration nights to the initial therapy upon randomization. Criteria for the remaining in the study were an AHI of at least 5/h and exclusion of a relevant PLMD syndrome (PLMI <10/h) or other relevant movement disorders during baseline PSG.

After baseline PSG on the following day, patients were investigated by a cardiac autonomic function test.

For patients who had been randomized to receive the MAD, titration with the MAD took place during the first of the two titration nights with an individually adjusted protrusion of up to 70 % of the possible maximum. If the AHI remained ≥10/h after the first titration night, the protrusion was individually increased, as recorded by a gauge by another 10 % of the patient’s maximum protrusion capacity during the second titration night.

For patients who had been randomized to receive CPAP therapy during the two titration nights, manual titration was performed to eliminate apneas, hypopneas, oxygen desaturations, and respiratory arousals.

After the three baseline PSG nights at T1, the patients were sent home for 12 weeks of continuous use of therapy during sleep with either MAD or CPAP. Afterward, the patients were invited to the sleep lab for another 3 consecutive nights without gap by PSG (T2).

For patients who had been randomized to receive the MAD as the following therapy, the MAD was individually produced and fitted to the patient by a dentist 1–2 weeks prior to the beginning of this therapy (T2).

During the first PSG at T2, patients were investigated while using the therapy that they had initially applied to measure the 12-week therapeutic effect on sleep.

After PSG on the following day, patients were again investigated by a cardiac autonomic function test to measure the 12-week therapeutic effect of the initial therapy on daytime cardiovascular functions.

The subsequent two PSGs of T2 served as titration nights for the following therapy mode and were performed as described for T1. After the three subsequent PSG nights at T2, patients were sent home for 12 weeks of continuous use of the following therapy. After this period, patients were invited to the sleep lab for a single night with PSG (T3). Patients were investigated by PSG to measure the therapeutic effect on sleep by the therapy that they had employed during the previous 12 weeks. After PSG on the following day, patients were investigated once again by a cardiac autonomic function test to measure the 12-week therapeutic effect on daytime cardiovascular functions.

#### Polysomnography

PSG was performed in a sleep lab certified by the German Sleep Society Board according to the American Academy of Sleep Medicine (AASM) guidelines by using an *Embla N7000* recorder (Embla systems, Broomfield, CO, USA).

Manual scoring of sleep resulted in the following parameters: percentage of sleep stages N1, N2, N3, and *R* of total sleep time (% TST); sleep latency (SL); wake time after sleep onset (WASO); and sleep efficiency (SE). Manual scoring of respiratory events during sleep enabled calculation of the apnea index (AI), the hypopnea index (HI), the AHI, and the oxygen desaturation index (ODI).

Scoring of sleep and respiratory events took place according to the AASM guidelines.

#### Cardiac autonomic function test

The cardiac autonomic function test was performed in the morning hours during wakefulness in a quiet room of the sleep lab. Patients were instructed not to use caffeine or vasoactive drugs during the morning of the test. Patients were studied in a 45° head-up position and were trained in metronomically controlled breathing at varying respiration frequencies, for a duration of 5 min in each case. Non-invasive continuous blood pressure was recorded with the Portapres^®^ system (Finapres Medical Systems, Amsterdam, The Netherlands). This method correlates well with intra-arterial blood pressure values and proved to be valid for time- and frequency-domain analysis of blood pressure variability and BRS [28]. In parallel, ECG (lead II), respiration with a thoracic effort belt, and nasal airflow were recorded using a PSG recorder (Embla systems, Broomfield, CO, USA). Subjects were instructed to breathe a) spontaneously, b) at a fixed rate of 6/min, c) at a fixed rate of 12/min, and d) with a fixed rate of 15/min. This protocol had likewise been used in earlier studies [29–31]. The fixed breathing rates were presented to the patients by a metronome. The sequence of these episodes of forced breathing, uniformly specified for every patient, included breaks of 2 min in which Riva-Rocci office blood pressure measurements were taken before raising the breathing rate.

The sampling rate was set to 200 Hz for the ECG and the continuous finger blood pressure signals and to 50 Hz for the respiration signals. The recording time duration for the four subsequent breathing modalities was 5 min each.

### Continuous heart rate and blood pressure analysis

The ECG and the continuous blood pressure signals recorded with the cardiac autonomic function test were analyzed using the MATLAB^®^ software (The Math-Works Inc., Natick, MA, USA), with algorithms as previously described [29, 32]. After offline filtering of 5-min signals using a Butterworth band pass (0.3–70 Hz) for each subsequent heartbeat, measurement of the following took place by peak-detection algorithms: the RR interval, the systolic blood pressure, and the diastolic blood pressure. Beat-to-beat mean blood pressure values were obtained by integration of continuous blood pressure values between subsequent heartbeats. Artifacts were thereafter removed and episodes of 3 min in which the breathing pattern was stable were selected for further analysis from the 5 min records. Interpolation of the time series then followed at 4 Hz by using a cubic spline interpolation algorithm.

In the time domain, we calculated the mean heart rate, the standard deviation of NN intervals (SDNN), and the root mean square of successive differences (RMSSD). In the frequency domain, fast Fourier transformation was applied to calculate power spectra of HRV and SBPV. For each of the two spectra, the low frequency (LF 0.04 to 0.15 Hz) and high frequency (HF 0.15 to 0.4 Hz) spectral bands were calculated.

For subjects in the supine position under controlled respiration without pharmacological or physical interventions, it can be assumed that the HRV HF band is primarily modulated by vagal activity, whereas the LF band reflects both sympathetic and vagal autonomic activity [33]. The LF/HF-HRV ratio was calculated as a surrogate for sympathovagal balance. An increase in the LF/HF-HRV ratio indicates a shift toward a sympathetic predominance in which higher sympathetic activity is causative as long as the HF component remains unchanged. SBPV components in the LF range are related to sympathetic activity, although this relationship is not specific. HF-SBPV component modulations are due to mechanical effects of respiration.

BRS is based on the permanent activity of the baroreflex feedback loop, in which amplitude fluctuations of blood pressure are transferred to corresponding heart rate changes. By use of spectral analysis of spontaneous heart and systolic blood pressure changes, it was possible to calculate spontaneous BRS by applying the square root of the ratio of RR interval and systolic blood pressure (SBP) power spectra. BRS values are expressed in milliseconds per millimeter of mercury (msec/mmHg).

This calculation was performed individually for the LF (0.04 to 0.15 Hz) and HF (0.15 to 0.4 Hz) spectral bands, which produced α-LF and α-HF [34] results. The squared coherence function (k2) was used to evaluate the statistical reliability of α-LF and α-HF. Data were further utilized if k2 exceeded 0.56 [35]. Under normal breathing conditions, α-LF shows the gain in the ratio of arterial pressure to RR interval in the spectral region not linked to breathing frequency. The α-HF component reflects the gain in the spectral region of the breathing frequency [34]. As a final point, α-tot was calculated as the mean of α-LF and α-HF and serves for evaluation of the overall baroreceptor gain [34].

### Statistics

For all statistical analysis, we set the alpha level at *p* < 0.05 and employed the IBM software SPSS^®^ Statistics, version 20.0 (IBM Corp., Armonk, NY, USA). Values are presented as mean ± standard deviation (SD). Data presented in figures indicate values for the 5th, 25th, 50th (median), 75th, and 95th percentiles.

To test for possible carryover effects of the therapy modes, the Mann–Whitney test was employed between sum values of sequence groups MAD–CPAP and CPAP–MAD.

To test for differences between measured values for baseline, 12 weeks of MAD therapy, and 12 weeks of CPAP therapy for parameters involving sleep, respiration, and the cardiac autonomic function test, the Friedman test was employed. Post-hoc analysis to identify significant pairs was performed by applying the Wilcoxon test.

To test for differences between MAD and CPAP treatment modalities, the Mann–Whitney test was used for difference values of the sequence groups MAD–CPAP and CPAP–MAD.

## Results

### Subject characteristics

Out of *n* = 84 patients with suspicion of OSA who were initially invited to participate, *n* = 48 were included in the study on the basis of inclusion and exclusion criteria. Reasons for exclusion were periodontitis (*n* = 20), phobia of dental work (*n* = 3), an AHI of <5/h (*n* = 5), the occurrence of a central sleep apnea syndrome (*n* = 1), and personal reasons (*n* = 7). After a period of 2 × 12 weeks, *n* = 40 patients (m = 33, w = 7) successfully completed the study. Causes for discontinuation of the study were insufficient compliance with CPAP therapy (*n* = 2), personal reasons (*n* = 2), intolerance of CPAP therapy (*n* = 3), and data loss due to technical failure (*n* = 1). Mean age was 49.5 ± 11.8 years and BMI was 28.3 ± 4.7 kg/m^2^. Mild sleep apnea (AHI 5 to 15/h) was determined in *n* = 3 patients, moderate sleep apnea (AHI >15 to 30/h) in *n* = 24, and severe sleep apnea (AHI >30/h) in *n* = 13. Mean ESS score was 9.2 ± 3.9, and a value of ESS ≥10—indicating an increased tendency to fall asleep—was established among half the patients. The average ISI score was 10.8 ± 4.9. Complaints of mild insomnia, expressed by an ISI score of 8 to 14, was seen in *n* = 22 patients, and moderate insomnia, with an ISI score 15 to 21, was observed in another *n* = 7 patients.

### Therapeutic effects of MAD and CPAP on PSG measures

No significant carryover effects on sleep structure or respiration were disclosed by PSG for sequence groups MAD–CPAP and CPAP–MAD.

#### Sleep structure

After 12 weeks of therapy, NREM sleep stage N1 was reduced by 7.7 %-TST for MAD (*p* < 0.01) and by 11.6 %-TST for CPAP (*p* < 0.01). Comparison of treatment modalities disclosed that the reduction was more pronounced after CPAP use than after MAD (*p* < 0.001). Rapid eye movement (REM) sleep increased by 2.3 %-TST for MAD (*p* < 0.01) and by 3.9 %-TST for CPAP (*p* < 0.01) in reference to baseline PSG values. Slow wave sleep (SWS) stage N3 likewise increased by 3.9 %-TST (*p* < 0.01) after 12 weeks of CPAP therapy, whereas for MAD, no significant changes were found. Comparison of CPAP and MAD results revealed no differences between treatment modalities with respect to sleep stages REM and N3. Parameters N2, WASO, SL, and SE remained unchanged with therapy in both treatment modalities. Also, comparison of CPAP and MAD results revealed no differences between treatment modalities for these parameters (Table 1).

**Table 1:** Effect of CPAP and MAD therapy on parameters of sleep structure from polysomnography (PSG)

Parameter	Baseline, *n* = 40	MAD, *n* = 40	CPAP, *n* = 40
N1 [% TST]	21.0 ± 13.0	13.3 ± 7.5*	9.4 ± 4.2*^,^**
N2 [% TST]	44.7 ± 10.2	47.2 ± 11.4	46.7 ± 13.8
N3 [% TST]	18.9 ± 10.0	21.3 ± 11.0	22.8 ± 11.7*
REM [% TST]	15.9 ± 5.6	18.2 ± 5.2*	19.9 ± 5.0*
WASO [min]	51.0 ± 34.9	44.2 ± 39.2	58.5 ± 46.9
TST [min]	400.3 ± 51.4	394.5 ± 59.4	400.5 ± 59.1
SE [%]	86.9 ± 7.9	88.2 ± 9.4	85.8 ± 11.6
SL [min]	15.9 ± 12.4	19.4 ± 19.7	20.2 ± 19.8

Values are means ± SD

*CPAP* continuous positive airway pressure therapy, *MAD* mandibular advancement therapy, *REM* rapid eye movement, *WASO* wake after sleep onset time, *SE* sleep efficiency, *SL* sleep latency

*p < 0.01 for comparison MAD vs. baseline or CPAP vs. baseline; **p < 0.001 for comparison CPAP vs. MAD

#### Respiratory parameters

Twelve weeks of treatment with MAD and with CPAP led with both modalities to changes in respiratory parameters.

Mean oxygen saturation values of 94.7 ± 1.5 % (range of 90.0 to 97.9 %) at baseline did not change after 12 weeks of MAD therapy (94.8 ± 1.2 %, range of 90.0 to 97.9 %), but after CPAP therapy (95.9 ± 1.1 %, range of 93.8 to 98.0 %) a reduction of 1.2 % (*p* < 0.001) became apparent. Comparison of CPAP with MAD disclosed significant difference between treatment modalities (*p* < 0.001). The ODI changed from 21.5 ± 14.8/h (range of 0.6 to 67.1/h) at baseline to 11.8 ± 11.4/h (range of 0.7 to 59.4/h) for MAD therapy (*p* < 0.001) and to 4.0 ± 6.5/h (range of 0.0 to 29.7/h) for CPAP therapy (*p* < 0.001). Comparison of CPAP to MAD revealed a significant difference between treatment modalities (*p* < 0.001).

**Fig. 2:**
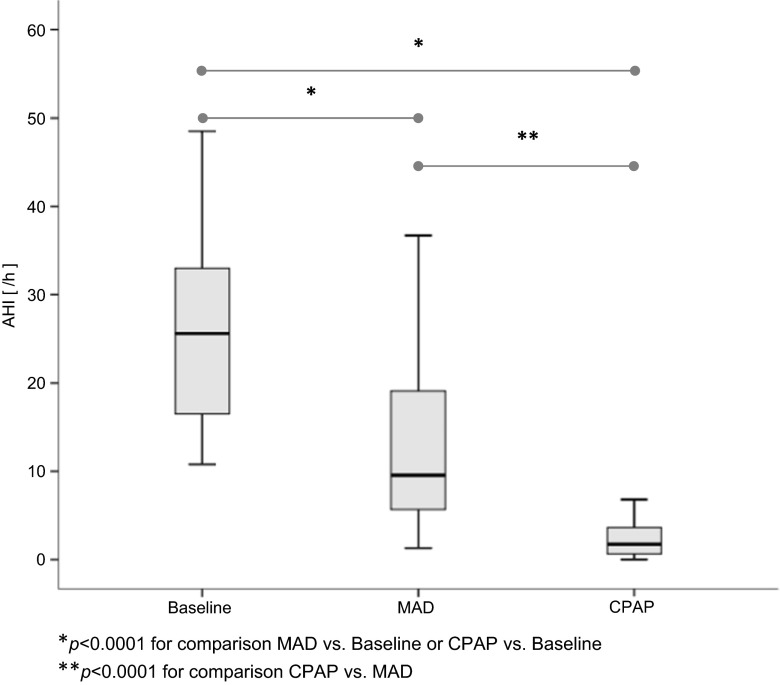
Effect of MAD and CPAP therapy on the apnea-hypopnea index (AHI) as determined by polysomnography (PSG)

The mean baseline AHI of 28.5 ± 16.5/h (range of 10.8 to 83.6/h) diminished with MAD treatment by 14.8/h (*p* < 0.001) to 13.7 ± 12.0/h (range of 1.3 … 59.6/h), whereas with CPAP treatment, a reduction by 25.0/h (*p* < 0.001) to 3.5 ± 5.2/h (range 0.0 … 23.6/h) became evident (Fig. 2). Separate analysis of apneas and hypopneas revealed for the AI a reduction from 17.8 ± 16.5/h (range of 0.3 … 23.6/h) at baseline to 6.8 ± 10.3/h (range of 0.0 … 55.1/h) for MAD therapy (*p* < 0.001) and to 1.1 ± 1.7/h (range of 0.0 … 8.2/h) for CPAP therapy (*p* < 0.001), whereas for the HI, a reduction from 11.1 ± 7.0/h (range of 0.0 … 37.6/h) at baseline to 7.0 ± 6.7/h (range of 0.7 … 34.4/h) for MAD therapy (p < 0.01) and to 2.4 ± 3.8/h (range of 0.0 … 16.2/h) for CPAP therapy (*p* < 0.001) was present. When comparing CPAP to MAD, there was a significant difference between treatment modalities for the AHI (*p* < 0.001), the AI (*p* < 0.001), and for the HI (*p* < 0.001). For treatment with MAD in *n* = 8 patients (20.0 %) and for CPAP in 33 patients (82.5 %), reduction in the number of breathing cessations to an AHI ≤5/h was observed. With assumption of a cutoff value of AHI ≤10/h for AHI reduction, for MAD therapy, a response thereto was established in *n* = 22 patients (55.0 %) and for CPAP therapy, in *n* = 37 patients (92.5 %).

### Therapeutic effects of MAD and CPAP on daytime cardiac autonomic function

Analysis of results from the cardiac autonomic modulation test performed during daytime with the various forced breathing patterns—i.e., a) spontaneously, b) with a fixed rate of 6/min, c) with a fixed rate of 12/min, and d) with a fixed rate of 15/min—revealed no significant carryover effects between sequence groups MAD–CPAP and CPAP–MAD.

#### Office blood pressure

Office systolic as well as diastolic blood pressure values measured by the Riva-Rocci method according to ESC/ESH guidelines before each of the four forced breathing sequences showed no differences after 12 weeks of MAD and CPAP therapy. Comparison of CPAP and MAD results revealed no differences between treatment modalities with respect to office blood pressure (Table 2).

**Table 2:** Effect of MAD and CPAP therapy on office blood pressure and on 3 min mean values of the daytime cardiac autonomic function test under conditions of spontaneous breathing, breathing at 6, 12, and 15/min

Breathing pattern	Spontaneous			6/min			12/min			15/min		
	Baseline	MAD	CPAP	Baseline	MAD	CPAP	Baseline	MAD	CPAP	Baseline	MAD	CPAP
Office blood pressure												
Riva Rocci-syst (mmHg)	121.9 ± 14.1	119.6 ± 12.6	119.6 ± 10.5	122.5 ± 14.8	117.0 ± 12.8	118.6 ± 11.1	122.9 ± 15.6	117.8 ± 13.2	118.3 ± 11.2	123.8 ± 16.0	121.1 ± 13.6	119.3 ± 10.37
Riva Rocci-diast (mmHg)	78.6 ± 9.3	77.8 ± 8.8	79.6 ± 7.4	79.9 ± 9.5	77.8 ± 9.0	79.3 ± 8.8	79.9 ± 16.0	78.5 ± 8.3	79.7 ± 8.5	80.7 ± 9.8	80.3 ± 9.0	80.1 ± 8.4
Time domain												
RR interval (ms)	934.3 ± 128.1	942.3 ± 98.3	931.7 ± 123.5	952.4 ± 123.2	958.4 ± 131.7	973.8 ± 164.1	940.8 ± 131.3	960.2 ± 107.2	948.2 ± 114.9	949.3 ± 126.9	957.3 ± 107.5	962.0 ± 106.7
SDNN (ms)	35.1 ± 18.1	36.1 ± 20.9	41.1 ± 19.7	56.2 ± 31.5	57.3 ± 30.5	60.8 ± 33.7	39.2 ± 15.0	39.6 ± 21.4	39.9 ± 21.4	37.0 ± 15.9	45.7 ± 26.1	45.3 ± 22.3
RMSSD (ms)	26.2 ± 20.7	26.4 ± 17.2	27.3 ± 19.1	39.3 ± 37.8	36.5 ± 23.7	41.9 ± 40.5	26.2 ± 15.4	30.4 ± 18.0 **	23.7 ± 19.8	26.5 ± 14.2	34.0 ± 22.2 **	30.2 ± 21.3
BP-syst (mmHg)	123.9 ± 20.3	119.2 ± 26.9	125.9 ± 20.7	123.9 ± 21.1	118.6 ± 21.7	119.6 ± 19.7	125.0 ± 22.5	121.6 ± 20.0	129.1 ± 25.4	133.6 ± 29.0	125.5 ± 16.5	124.8 ± 24.0
BP-mean (mmHg)	87.3 ± 12.7	79.5 ± 17.4 **	81.0 ± 11.7 **	86.2 ± 14.5	79.5 ± 15.5 **	77.8 ± 12.6 *	86.5 ± 13.2	81.4 ± 13.6 **	84.6 ± 16.7	92.3 ± 21.9	82.6 ± 9.20 **	82.9 ± 17.8 **
BP-diast (mmHg)	69.3 ± 13.3	60.5 ± 17.7 *	60.9 ± 12.1 *	68.8 ± 14.1	61.6 ± 15.6 *	59.4 ± 11.8 *	69.1 ± 12.2	62.6 ± 13.2 **	65.4 ± 15.4	73.9 ± 19.9	63.7 ± 9.38 **	64.2 ± 16.5 **
HRV												
LF (ms^2^)	26.6 ± 29.5	32.4 ± 59.7	37.4 ± 61.8	131.7 ± 133.7	134.1 ± 126.9	157.0 ± 171.3	22.6 ± 19.3	28.8 ± 43.0	21.6 ± 19.6	22.1 ± 23.1	38.9 ± 55.6	27.8 ± 31.0
HF (ms^2^)	15.9 ± 22.7	22.3 ± 30.3	19.0 ± 31.5	26.1 ± 66.3	18.0 ± 22.5	28.6 ± 61.3	24.0 ± 31.0	33.6 ± 37.9 *	25.5 ± 40.3	16.5 ± 16.0	23.4 ± 25.3 **	20.5 ± 31.8
LF/HF ratio	2.5 ± 3.1	2.7 ± 4.4	3.3 ± 3.5	13.2 ± 13.3	14.1 ± 10.0	13.6 ± 11.5	1.8 ± 1.4	1.8 ± 2.4	1.8 ± 1.8	1.0 ± 1.1	1.9 ± 1.9	2.3 ± 2.1
SBPV												
LF (mmHg^2^)	0.48 ± 0.47	0.48 ± 0.46	0.59 ± 0.46	0.80 ± 0.58	0.92 ± 0.67	1.12 ± 0.59 *	0.35 ± 0.19	0.40 ± 0.43	0.36 ± 0.23	0.41 ± 0.30	0.49 ± 0.48	0.39 ± 0.38
HF (mmHg^2^)	0.22 ± 0.20	0.23 ± 0.21	0.16 ± 0.11	0.06 ± 0.04	0.07 ± 0.04	0.09 ± 0.09	0.17 ± 0.11	0.19 ± 0.12	0.18 ± 0.13	0.16 ± 0.15	0.15 ± 0.10	0.15 ± 0.11
LF/HF ratio	4.6 ± 6.8	3.0 ± 4.7	7.2 ± 10.5	15.9 ± 11.0	18.1 ± 12.5	20.7 ± 11.9 **	2.7 ± 1.9	2.4 ± 1.8	3.2 ± 2.6	4.0 ± 2.9	4.0 ± 3.3	5.0 ± 6.8
BRS												
α-LF (ms/mmHg)	7.1 ± 4.0	6.7 ± 3.8	6.4 ± 2.8	11.4 ± 5.0	11.3 ± 5.7	10.4 ± 5.1	7.9 ± 3.2	7.9 ± 4.0	7.3 ± 4.3	7.5 ± 3.1	8.5 ± 6.6	8.4 ± 5.0
α-HF (ms/mmHg)	9.3 ± 6.8	9.1 ± 8.0	9.1 ± 7.0	15.2 ± 10.9	13.9 ± 9.8	14.5 ± 11.3	10.7 ± 6.4	12.0 ± 8.3	11.1 ± 9.1	13.6 ± 12.4	12.0 ± 8.7	11.7 ± 7.2
α-tot (ms/mmHg)	8.2 ± 5.1	7.9 ± 5.3	7.8 ± 4.5	13.3 ± 7.6	12.6 ± 7.4	12.4 ± 8.0	9.3 ± 4.1	9.9 ± 6.0	9.2 ± 6.3	10.6 ± 7.26	10.2 ± 7.3	10.0 ± 5.8

Values are means ± SD

*CPAP* continuous positive airway pressure therapy, *MAD* mandibular advancement therapy, *SDNN* standard deviation of NN intervals, *RMSSD* root mean square of successive differences

*p < 0.05 for comparison CPAP vs. baseline or MAD vs. baseline; **trend (*p* < 0.1) for comparison CPAP vs. baseline or MAD vs. baseline

#### Time domain values: RR interval, SDNN, RMSSD, and blood pressure

In comparison with baseline, RR interval values as well as SDNN values did not differ with either MAD therapy or CPAP therapy. Again, comparison of CPAP with MAD results disclosed no differences between treatment modalities. These results were consistently found for all four applied respiratory protocols. For RMSSD, a trend (*p* < 0.1) for higher values after 12 weeks of therapy with MAD were found for forced breathing rates of 12 and for 15/min. Comparison of CPAP and MAD results revealed no differences between treatment modalities.

With regard to mean systolic blood pressure, neither MAD nor CPAP values differed from baseline values, and CPAP and MAD comparisons showed no differences between treatment modalities.

With MAD therapy, the mean blood pressure showed a trend for lower values in all four applied forced breathing patterns. With CPAP therapy, a significant fall became evident in mean blood pressure under the condition of 6/min and of a trend toward lower values under conditions of spontaneous breathing and of 15/min. Comparison of CPAP and MAD results disclosed no differences between treatment modalities (Table 2).

**Fig. 3:**
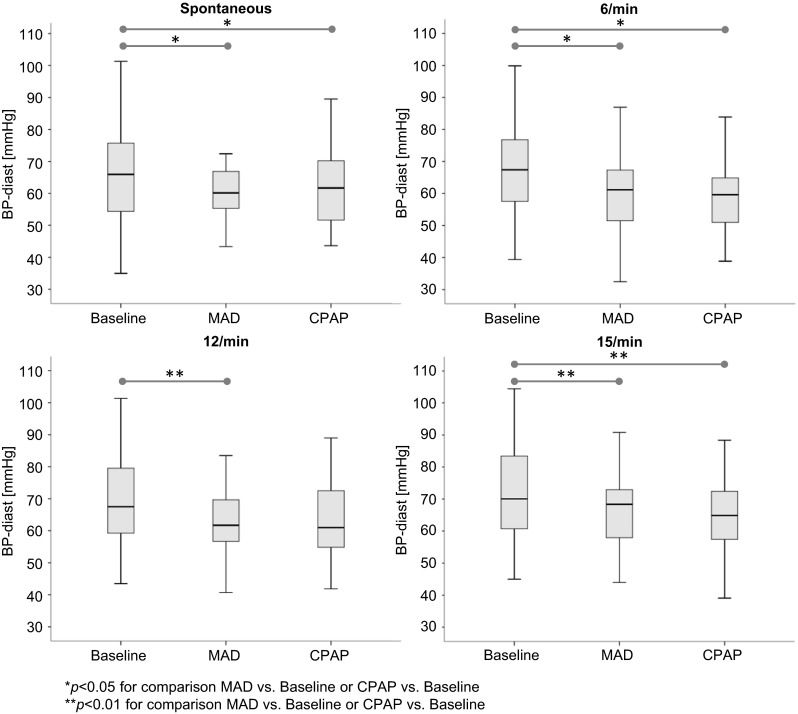
Effect of MAD and CPAP therapy on three-minute mean values of continuous diastolic blood pressure recordings (BP-diast) under conditions of spontaneous breathing, breathing at 6/min, 12/min, and 15/min, from the daytime cardiac autonomic function test

With MAD therapy, diastolic blood pressure was significantly reduced by an average of 8.9 mmHg for spontaneous breathing (*p* < 0.05). Values were likewise reduced by an average of 7.3 mmHg for 6/min (*p* < 0.05), and a trend for lower values became evident for 12/min as well as for 15/min. With CPAP therapy, diastolic blood pressure was significantly reduced by an average of 8.4 mmHg for spontaneous breathing (*p* < 0.05) as well as on average by 9.5 mmHg for 6/min (*p* < 0.05). A trend for lower values was apparent for 15/min. Comparison of CPAP with MAD showed no differences between treatment modalities (Table 2, Fig. 3).

#### Frequency domain values: HRV, SBPV, and BRS

In comparison to baseline, mean HRV-LF values as well as mean HRV-LF/HF values did not differ with either MAD or CPAP therapy. These results consistently appeared for all four applied breathing patterns. After 12 weeks of MAD therapy, the HRV-HF component increased by 9.59 ms^2^ for 12/min (*p* < 0.05) and showed a trend toward higher values for 15/min. Only at forced breathing at 6/min after use of CPAP did SBPV-LF values increase by 0.324 mmHg^2^ (*p* < 0.05); SBPV-LF/HF values demonstrated a trend toward higher values.

In comparison to baseline mean values of SBPV-HF, α-LF, α-HF, and α-tot differed neither with MAD therapy nor with CPAP therapy. These results were consistently found for all four applied breathing patterns.

Comparison of CPAP with MAD revealed that there were no differences between treatment modalities for all parameters of HRV, SBPV, and BRS (Table 2).

## Discussion

For the first time, the effects of 12-week CPAP and 12-week MAD therapy on cardiovascular and autonomic parameters during daytime were investigated, under conditions of controlled breathing. Both CPAP and MAD therapy led to changes in blood pressure in patients with mild to severe OSA. In addition, changes in HRV values were observed for therapy with MAD. Both CPAP and MAD therapy substantially eliminated breathing cessations, with an advantage for CPAP treatment. These findings became evident regardless of the definition of efficacy and the severity of OSA and are in line with previous studies dealing with this topic [12–14]. Additionally, CPAP improves REM and SWS sleep, while MAD shows a significant effect on REM sleep only. No effects of therapy were detected for total sleep time, wake time after sleep onset, or sleep efficiency.

With regard to parameters for vagal autonomic activity, the HRV-HF component increased with 3 months of MAD use, but not with CPAP. Similarly, a trend toward greater HRV RMSSD values became apparent with MAD use. Analysis of continuous blood pressure measurements from the cardiovascular autonomic function test revealed changes in mean and diastolic pressures, but not in systolic blood pressure. Diastolic blood pressure declined markedly with both CPAP and MAD treatment. Mean blood pressure benefited from CPAP treatment only.

No effects were detected on daytime blood pressure as measured with the Riva-Rocci method.

Analysis of autonomic activity in the frequency domain showed no effects with either treatment modality. Merely a minor effect on HRV-HF after MAD and on SBPV-LF after CPAP was detected after various breathing frequencies. No effects on BRS were found.

We evidenced that both treatment options may improve daytime blood pressure, especially diastolic. Our primary finding was accordingly that MAD is as effective as CPAP in improving parameters of daytime cardiac autonomic modulation, especially blood pressure.

This finding is surprising since the reduction of AHI by MAD is less than with CPAP. With respect to efficiency, the results of our study are in agreement with previous studies [15–20, 23]. We did not establish an exclusion criterion related to the AHI. We studied patients with a mean AHI of 28.6 ± 16.5/h (min 10.8/h, max 83.6/h), which is comparable to the other studies cited. Engleman et al. [23] also included severe apneics without restriction, whereas Aarab et al. [15], Ferguson et al. [17], Gagnadoux et al. [18], and Lam et al. [21] limited the AHI threshold to 40 to 60 events per hour of sleep.

We recruited only *n* = 13 patients with severe sleep apnea, which may be related to the fact that we are a specialized university center with more co-morbid patients than in an outpatient sleep center. Although we did not treat many severe patients, it is important to note that the higher the AHI, the higher the effect of MAD on the AHI is. In mild sleep apnea patients, we found a decline of AHI by about only 40 %; in moderate patients, by 43 %; and in severe patients, by 60 %. It is therefore evidently the case that, despite primary recommendation of MAD for mild to moderate OSA [2, 22], it may also offer benefits for a subgroup of patients with severe OSA. Some additional predictors may be supine-predominant OSA, younger age, female gender, less obesity, and certain craniofacial features [36].

The effect on sleep is comparable in both treatment options and is limited to an improvement in REM sleep for MAD and CPAP, and in SWS sleep for CPAP only, which highlights another advantage of CPAP therapy. Although such benefits are not dramatic, this aspect should be kept in mind when selecting patients for a special kind of treatment. On the other hand, it is necessary to note that the amount of SWS before treatment was within normal ranges—which limits a possible SWS rebound. REM sleep was reduced during the baseline night, which justified expectation of a treatment effect.

With regard to cardiac autonomic modulation, some effect has been shown until now only for CPAP [5, 8]. HRV is apparently a prominent marker for measuring the effect of CPAP on the autonomic system [5, 8], but some authors also reported a positive effect of CPAP on BRS [9, 10]. These studies, however, are not comparable owing to methodological differences involving time points at which measures were taken, methods of detection, analysis methods, and duration of treatment.

We determined such effects in the present study as well. We could not detect blood pressure changes at daytime as objectified with the standard Riva-Rocci measurement, but we detected blood pressure changes with the Portapres method. These changes were considerable. A blood pressure change of about 7 to 9 mmHg is clinically significant in comparison to the changes reported for CPAP treatment, which are much lower [4]. In addition, these results are in line with data from Phillips et al. [13] who also reported a change in diastolic blood pressure after MAD treatment, and from Gotsopoulos et al. [24] who reported a decrease in the 24 ambulatory blood pressure monitoring (ABPM) mean after 4 weeks of MAD treatment in comparison to sham MAD. Barnes et al. [16] also found a decline after 3 months of treatment in diastolic ABPM blood pressure, but only at nighttime in comparison to CPAP and to placebo. In general, for CPAP treatment, changes in systolic as well as in diastolic blood pressure were reported for daytime as well as for nighttime, but with low effect magnitudes [4]. The effect is more pronounced in patients with higher blood pressure and in patients with resistant hypertension [4]. In our study, we did not include patients with resistant hypertension. We included three patients who were being treated with antihypertensive medication.

The effects determined in our study would have possibly been even more pronounced if we had included patients with elevated blood pressure or resistant hypertension. A mean RR of 122.8/79.7 mmHg at baseline indicated that the patients were within normal blood pressure ranges or slightly higher.

It is also worthy of note, however, that the ambulatory ABPM is not comparable to our short-time series measurement. Second, the influence of breathing on ABPM and on our time series protocol can be different. We consequently conducted a special breathing protocol with fixed breathing frequencies. This allows a standardized comparison between CPAP and MAD, but not with data evaluated without control of respiration.

RMSSD improvement with the 3-month MAD treatment also represents the positive effect of treatment on autonomic tone. Nevertheless, it remains unclear why there are no significant changes after CPAP and why we could detect the changes with only two breathing frequencies (12/min and 15/min).

Surprisingly, BRS did not change at daytime after 3 months of both CPAP and MAD therapy. In addition, only small changes were seen in HRV-HF (with MAD) and in SBPV-LF (with CPAP). Although the changes in HRV and SBPV indicate positive treatment effects, it should be considered that our patients were without elevated cardiovascular risk, and no marked elevations of HRV, BPV, or BRS were present upon selection inclusion. These circumstances do not favor either therapy; they rather indicate that changes in these parameters during daytime and an improvement in these parameters with therapy were evident presumably only in patients with severe sleep apnea or in patients with severe co-morbidities. Distinguishing between OSA severity groups and/or occurrence of cardiovascular morbidities was outside the scope of this trial. In total, only *n* = 13 patients (33 %) suffered from severe sleep apnea and were free of co-morbidities. This number is low to obtain statistical meaningful results. This should be the subject for future investigation. Furthermore, it could be assumed that there may be acute changes during the night due to therapy.

### MAD technique issues

The results of our study are specific for the device we used. It must be noted that different MAD devices are not comparable with respect to efficacy and in accordance with AHI and type of protrusion [37]. We used a custom-made individual bimaxillary appliance, the same that Phillips et al. [13] used.

In contrast to CPAP treatment—for which we assume that device characteristics are similar when the same pressure is applied—there are evidently relevant MAD device differences [37].

In addition, a sufficient number of healthy teeth for anchorage are an important issue in selecting patients for treatment with MAD. Whether patients with sufficient teeth differ from other sleep apnea patients has not yet been clarified. When recruiting patients for the study, many could not be included owing to dental decay, morbid teeth, or an insufficient number of teeth. Occasionally, this makes fitting a patient with a MAD treatment more difficult and time consuming than the CPAP treatment. Well-organized co-operation with educated and experienced dentists is necessary to realize a patient flow without many dropouts.

### Study limitations

One limitation of this trial is that objective wear-time sensors were not available; such devices were unfortunately not available for the MADs used for this trial. For CPAP it is reported that compliance rates may influence changes in cardiac autonomic modulation [8] due to therapy; therefore, there is a need to fit MADs with wear-time sensors. Recent developments in oral appliances allow for such measures, for example, by enabling installation of an embedded micro sensor thermometer [38]. To promote good and comparable compliance rates in both treatment arms in the present trial, study participants were seriously advised to use the therapy for at least 6 h per night and for at least 6 days per week. Inadequate compliance with the instructions resulted in *n* = 3 dropouts during the course of the study.

Another limitation of this crossover study is that the change of treatment modality from MAD to CPAP or CPAP to MAD was not accompanied by a washout period, i.e., no therapy for a number of days. A recently published review by Vroegop et al. [39] concluded that there is some evidence that CPAP washout exists for some days in patients, although the intensity and duration remain unclear. Therefore, some influence of the preceding CPAP therapy on the subsequent titration to MAD could not be excluded. Comparison of therapy sequences MAD–CPAP with CPAP–MAD by statistical analysis, however, revealed no significant difference in results. After 12 weeks of therapy, this effect could consequently be neglected. For design of future crossover studies on therapeutic interventions for OSA, a washout period should be considered.

In conclusion, 12 weeks with MAD and with CPAP lead to changes in cardiac autonomic modulation, in particular, by lowering diastolic blood pressure. At the same time, both therapy modalities resulted in significant reductions in the number of breathing cessations, with greater significance in CPAP.
